# Supplementary material for: Genome-Wide Identification and Transcriptional Expression Profiles of PP2C in the Barley (Hordeum vulgare L.) Pan-Genome
Source: Genes (Basel). 2022 May 7;13(5):834. doi: 10.3390/genes13050834 (PMC9140614; doi:10.3390/genes13050834)
Supplement: Supplementary file 1 [file genes-13-00834-s001.zip › Supplementary Figures.pdf]

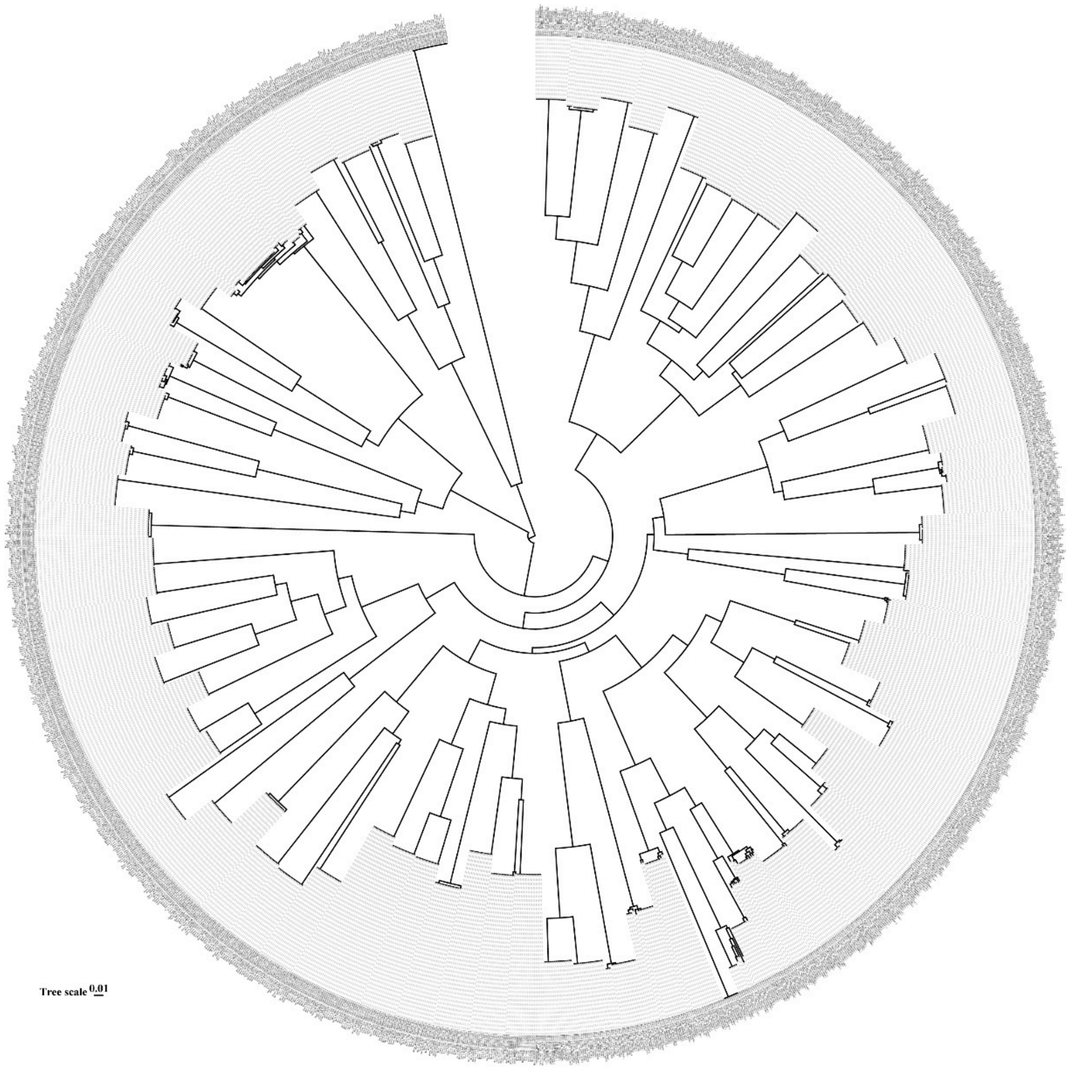

Figure S1. A phylogenetic ML-tree using all 1656 HvPP2C sequences

Logo	E-value	Sites	Width
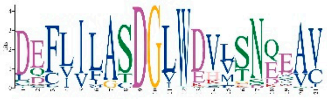	2.2e-971	83	29
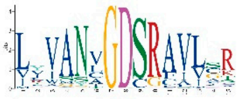	3.4e-557	85	15
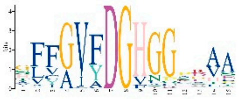	9.9e-507	82	15
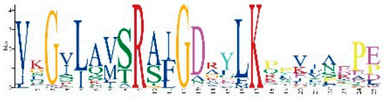	6.2e-454	48	25
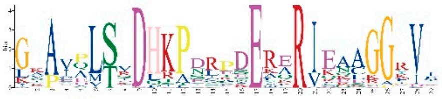	5.6e-515	48	29
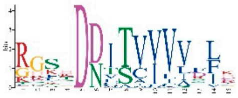	1.3e-395	79	15
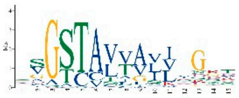	4.6e-238	84	15
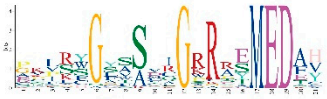	1.8e-163	30	21
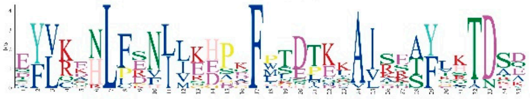	6.4e-154	16	35
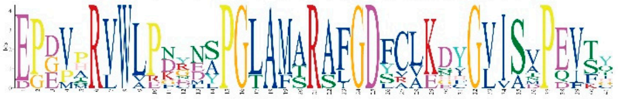	2.5e-144	10	41

Figure S2. The width, sites, and E-value and sequence logo of conserved motif of Morex HvPP2C proteins.

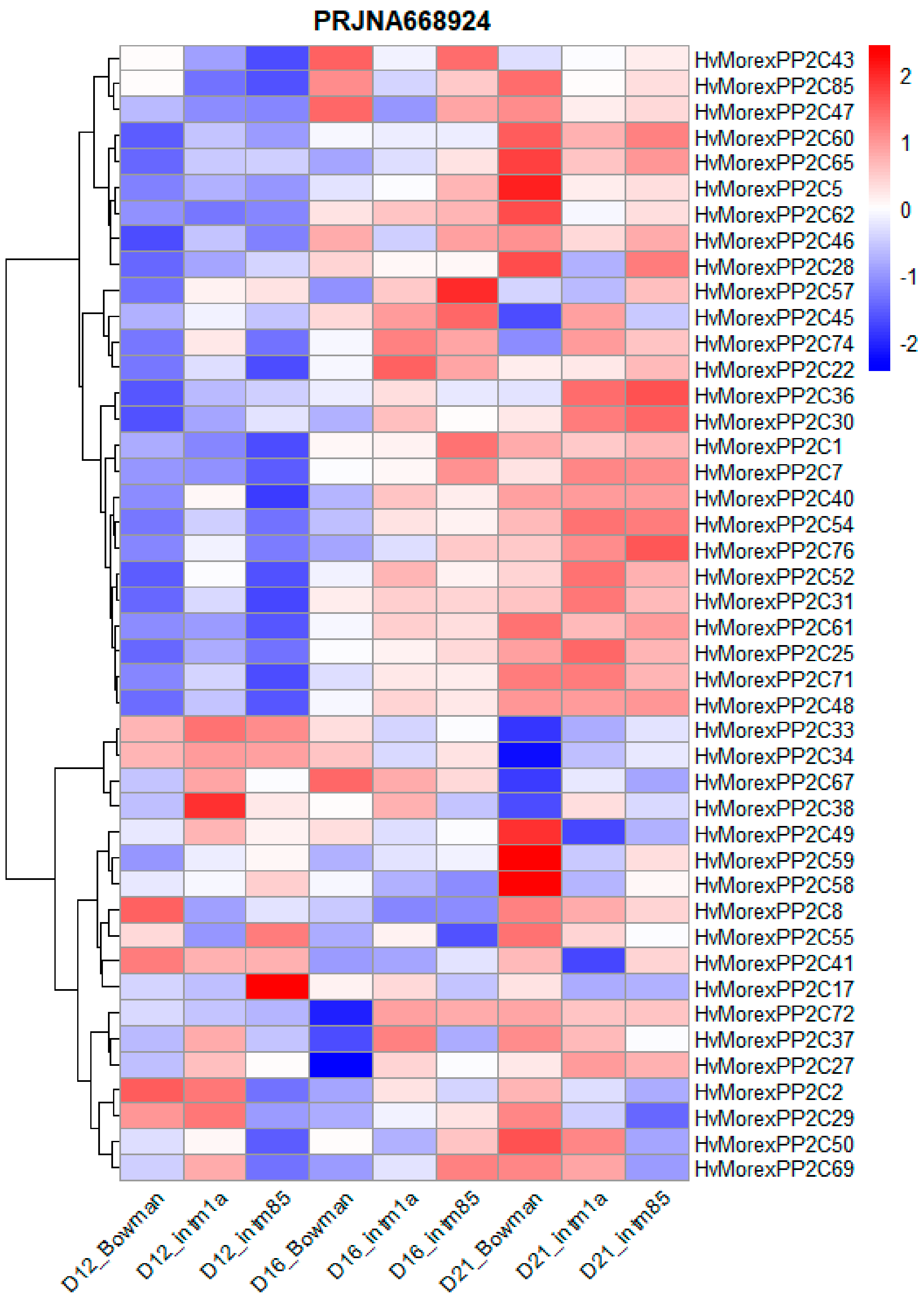

Figure S3. Expression profiles of barley PP2Cs during inflorescence development. Log2 based fold change was used to create the heatmap. Fold changes in gene expression are shown in color as the scale.

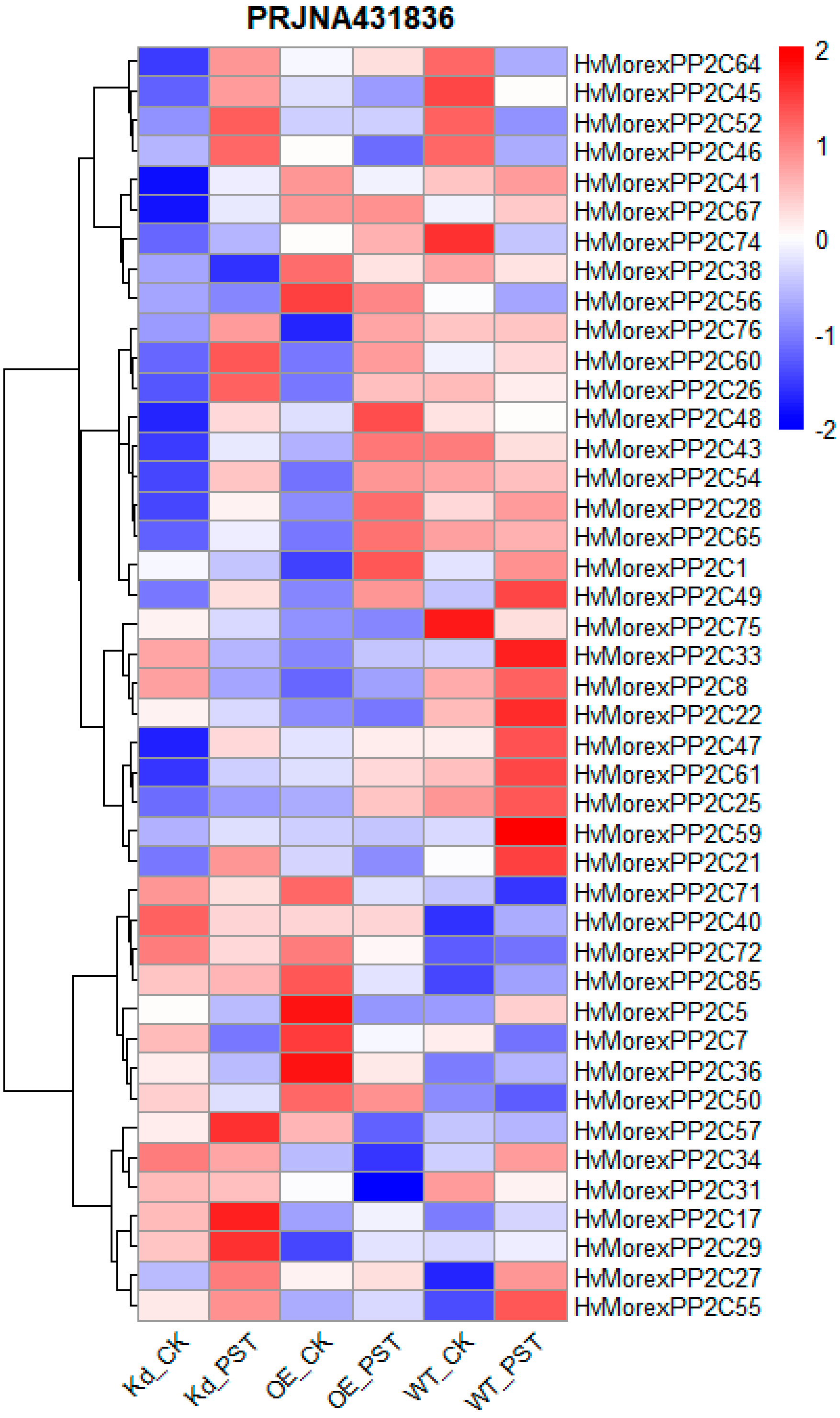

Figure S4. Expression profiles of barley PP2Cs during their NPR1-Mediated acquired resistance (AR) triggered by Pseudomonas syringae pv. tomato DC3000.

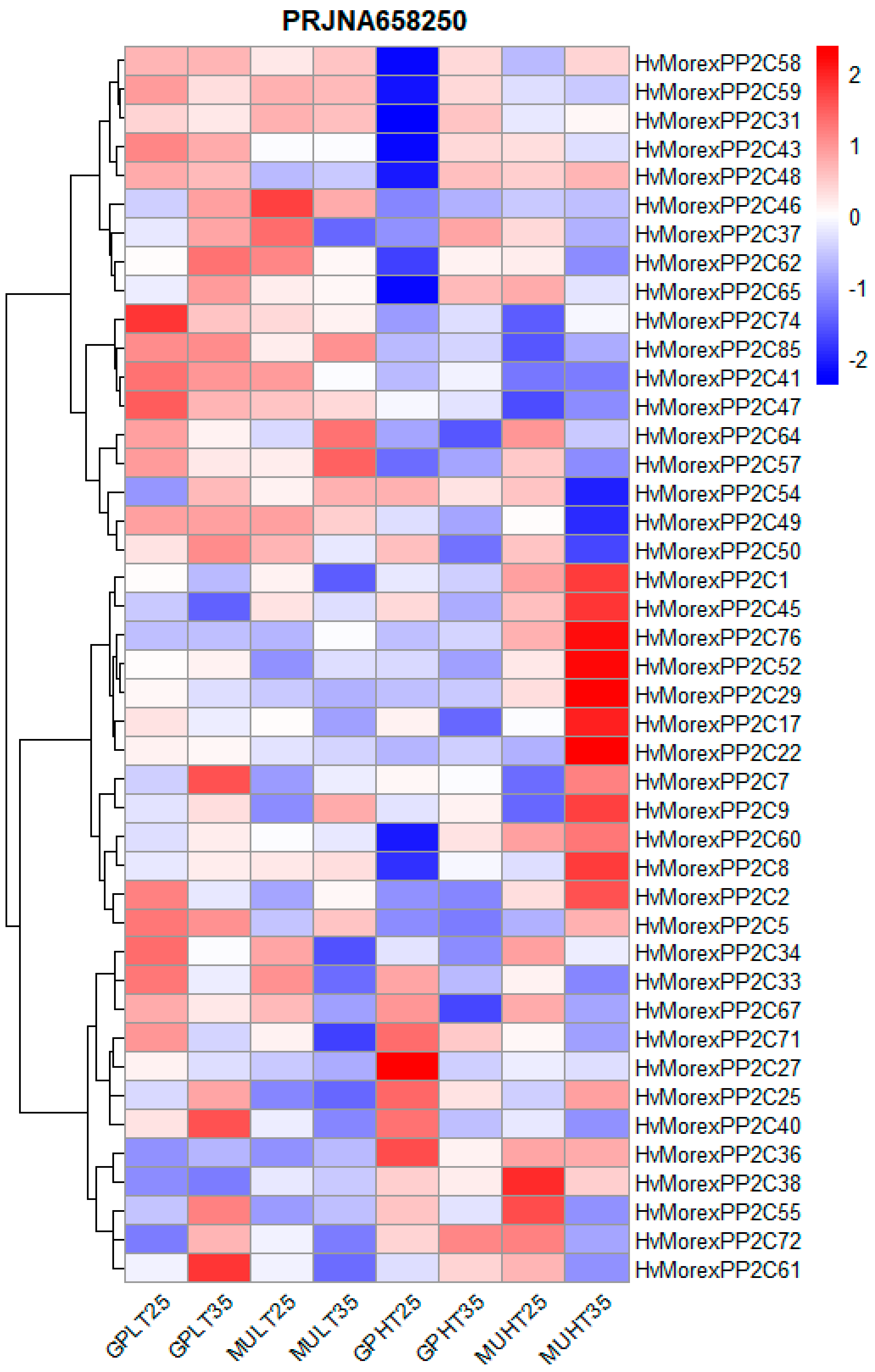

Figure S5. Expression profiles of barley PP2Cs in response to high ambient temperatures.

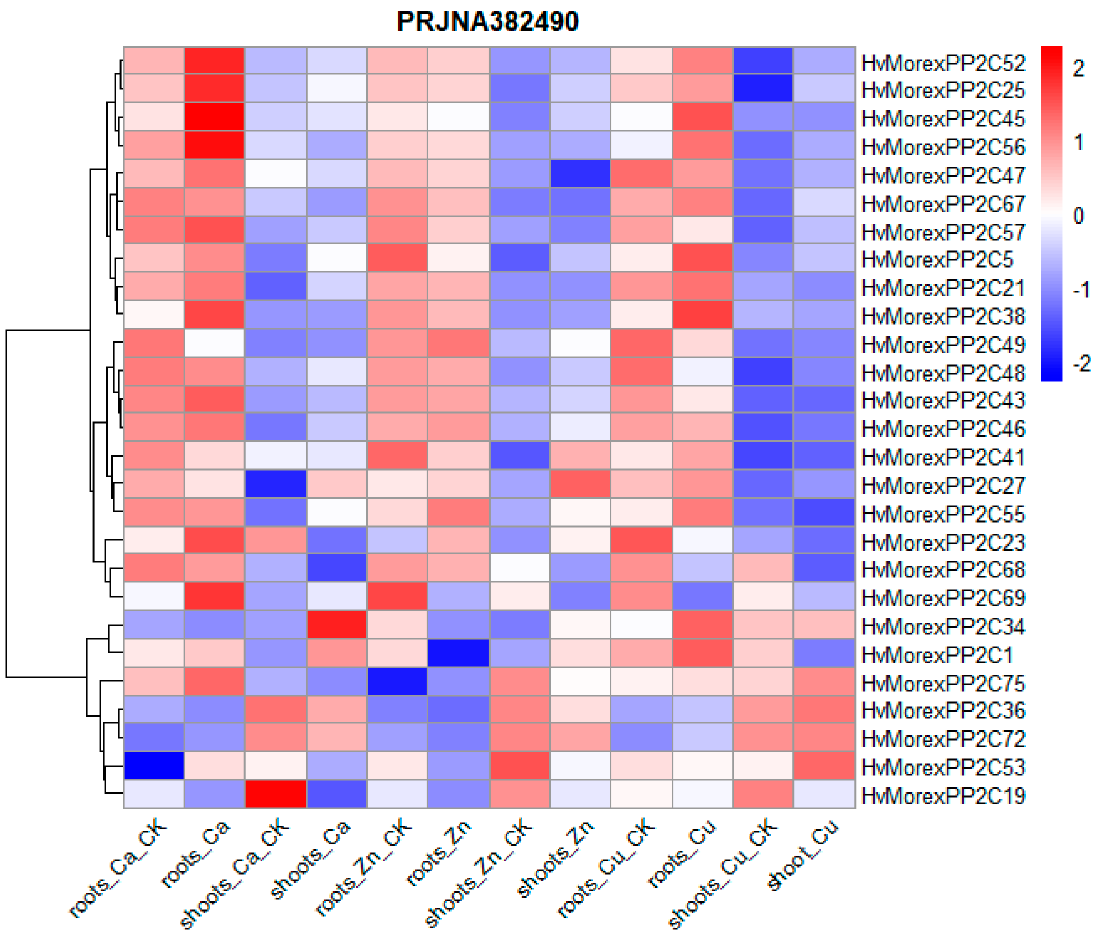

Figure S6. Expression profiles of barley PP2Cs in response to heavy metal stress.

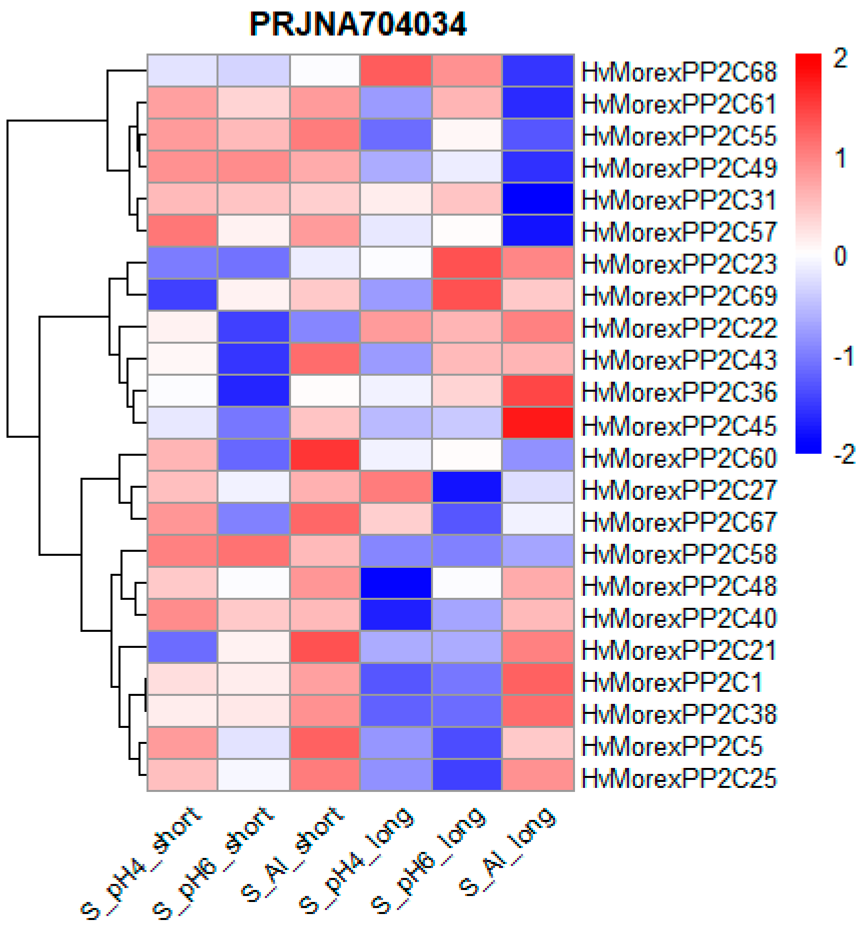

Figure S7. Expression profiles of barley PP2Cs in response to Aluminum and low pH stresses.

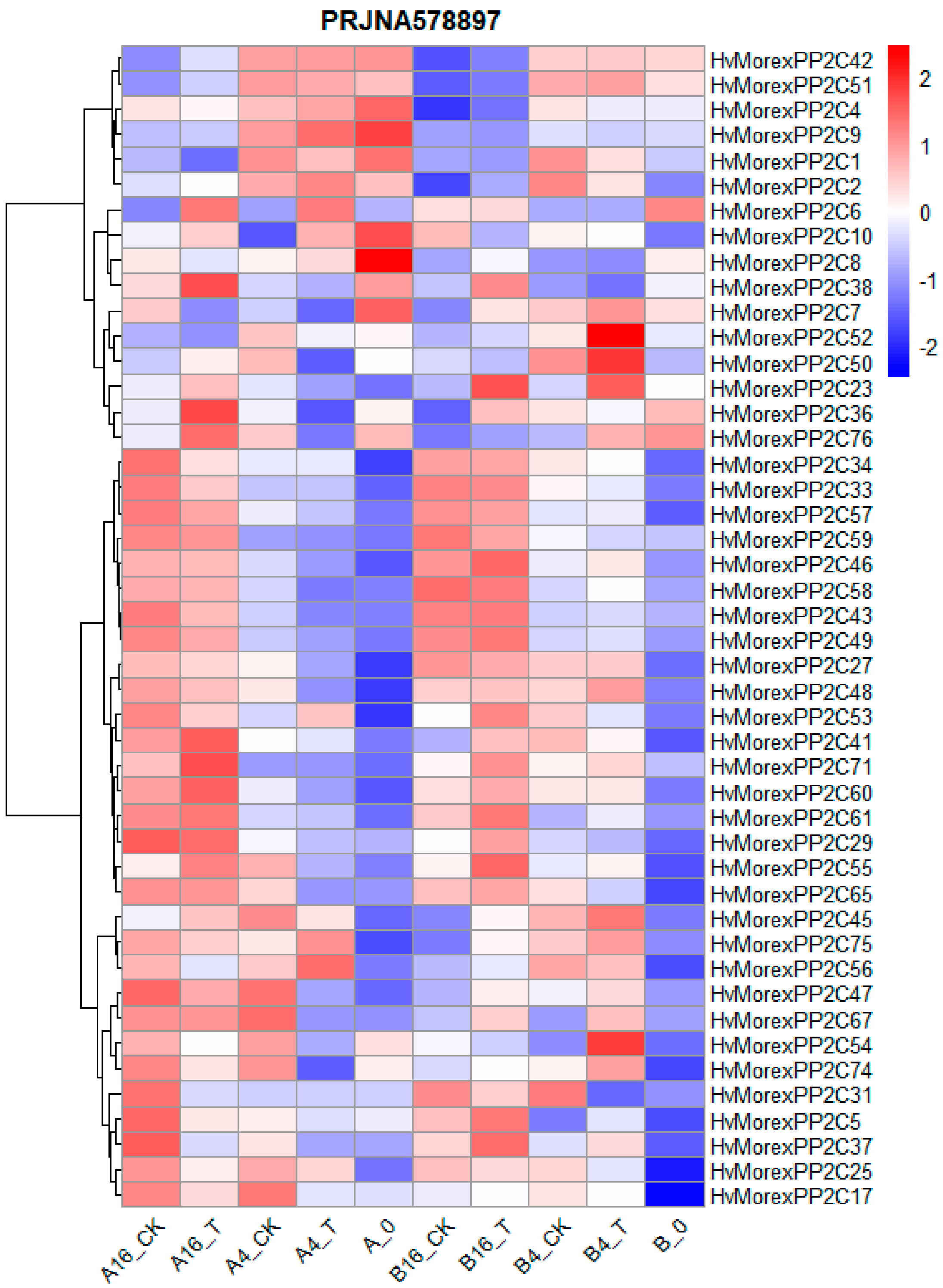

Figure S8. Expression profiles of barley PP2Cs in response to salt stress.
